# Analysis of scene-guided camera assistance in transaxillary gasless endoscopic thyroidectomy: a minor improvement in operative technique

**DOI:** 10.3389/fendo.2023.1146336

**Published:** 2023-04-19

**Authors:** Baihui Sun, Shitong Yu, Junna Ge, Zhicheng Zhang, Weisheng Chen, Zhigang Wei, Tingting Li, Shangtong Lei

**Affiliations:** Department of General Surgery & Guangdong Provincial Key Laboratory of Precision Medicine for Gastrointestinal Tumor, Nanfang Hospital, The First School of Clinical Medicine, Southern Medical University, Guangzhou, Guangdong, China

**Keywords:** transaxillary gasless endoscopic thyroidectomy, scene-guided camera assistance, camera holders, thyroid carcinoma, operative technique

## Abstract

**Background:**

Transaxillary gasless endoscopic thyroidectomy (TGET) is a widely performed operation, but its side view angle and instrument interference have caused concerns for most surgical groups. The aim of this study was to introduce scene-guided camera assistance (SGA) and analyze its role in facilitating TGET.

**Methods:**

We put forward key points for camera holders, including one pivot, two positions, and three planes, and separated TGET operations into five parts. We also established the view angle for each part of the operation for the camera holder to follow. Then, we reviewed 416 patients who underwent TGET with or without SGA and analyzed their demographic characteristics, operative outcomes, pathologic outcomes, and early complications.

**Results:**

The TGET and TGET-SGA groups were similar in terms of age, sex ratio, height, weight, tumor size, Hashimoto’s thyroiditis ratio, and cN1 ratio. The operation time and postoperative hospital stay were significantly longer in the TGET group than in the TGET-SGA group (114.43 ± 17.20 minutes vs. 101.82 ± 19.39 minutes and 3.16 ± 0.77 days vs. 2.16 ± 0.55 days, respectively, *P < 0.001*). The account of retrieved lymph nodes was less in the TGET group than in the TGET-SGA group (5.61 ± 4.27 vs. 6.57 ± 4.96, *P = 0.038*).

**Conclusion:**

SGA provided guidance for camera holders, and the data showed that it was an improvement for TGET operations.

## Introduction

Papillary thyroid cancer is a malignant disease whose incidence is increasing. It has drawn a great deal of attention from the public in China and worldwide (1). Since the majority of patients suffering from papillary thyroid cancer are females who have significant aesthetic concerns, endoscopic thyroidectomy through various remote-access approaches can be performed to avoid neck scars.

Transaxillary gasless endoscopic thyroidectomy (TGET) has been widely performed throughout Asia for a few decades (2). A systematic review concluded that TGET was a feasible and safe procedure despite of the disadvantage of a longer operation time (3). Our previous study showed the same pros and cons likewise (4). As a widely used procedure, TGET has a side-angle view which requires a perspective conversion from the traditional positions of the recurrent laryngeal nerve and parathyroid gland in conventional open thyroidectomy (5). Moreover, interference between the endoscopic lens and surgical instruments, which share one working space, is another obstacle that contributes to a longer operation time.

In order to minimize interference and improve the viewing experience of TGET, we intend to introduce a scene-guided camera assistance (SGA) approach to this operation. We standardized the camera’s positions and view angles, instructed assistants on how to assist during surgery, and analyzed the improving results of this assistance using a retrospective study. We analyzed the clinical outcomes of 416 TGETs performed by a single surgeon in the management of patients with papillary thyroid cancer at a single institution. Regarding the application of SGA as a variate, we divided these operations into TGET and TGET-SGA groups. Hopefully, our study will improve the viewing experience of endoscopic surgery novices.

## Materials and methods

### Patient enrollment and data collection

A total of 416 patients at Nanfang Hospital of Southern Medical University from January 2021 to June 2022 were enrolled in this retrospective study. The inclusion criteria were as follows: (1) diagnosis of papillary thyroid cancer; (2) tumor ≤ 2 cm in greatest dimension and limited to unilateral lobe of the thyroid; (3) no preoperational proof of locoregional lymph node metastasis (cN0) or only metastasis to unilateral level VI lymph nodes (cN1); and (4) no distant metastasis. The exclusion criteria were as follows: (1) patients who required total thyroidectomy; (2) patients who required lateral neck dissection; and (3) patients who had previously undergone cervical surgery or radiotherapy. All patients underwent lobectomy and isthmusectomy with central neck dissection by either TGET or TGET-SGA. The collected data included demographic characteristics, operative outcomes, pathologic outcomes, and early complications. This study was approved by the Ethics Committee of Nanfang Hospital of Southern Medical University, and written informed consent was obtained from the enrolled patients.

### Operation procedure under scene-guided camera assistance

Scene-guided camera assistance (SGA) went through all procedures during TGET surgery. It was a standardization on operation preparations, incision design, key pointes for camera holders, assistance during thyroidectomy and central neck dissection.

#### Operation preparations

**Figure 1:**
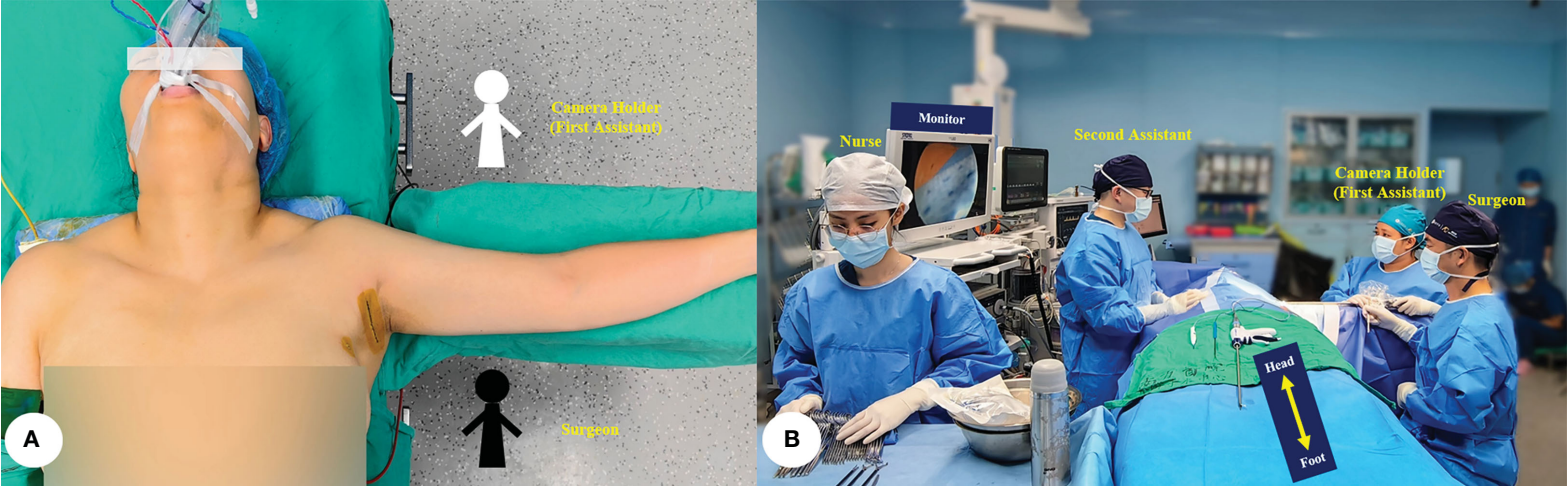
**(A)** The position of the patient. **(B)** The locations of operation equipment and stuff.

After general anesthesia with endotracheal intubation, the patient was placed in a supine position with a pad under the neck for extension. The arm on the lesion side was naturally abducted. The first assistant, also known as the camera holder, and the surgeon sat by the patient’s abducted arm, with the assistant closer to the patient’s head and the surgeon toward the patient’s foot. During the entire operation, the first assistant should hold the camera with both hands and gently lean both forearms on the patient’s arm as an extra pivot to help stabilize the view in the surgical area (**Figure 1**).

#### Incision design and working space creation

**Figure 2:**
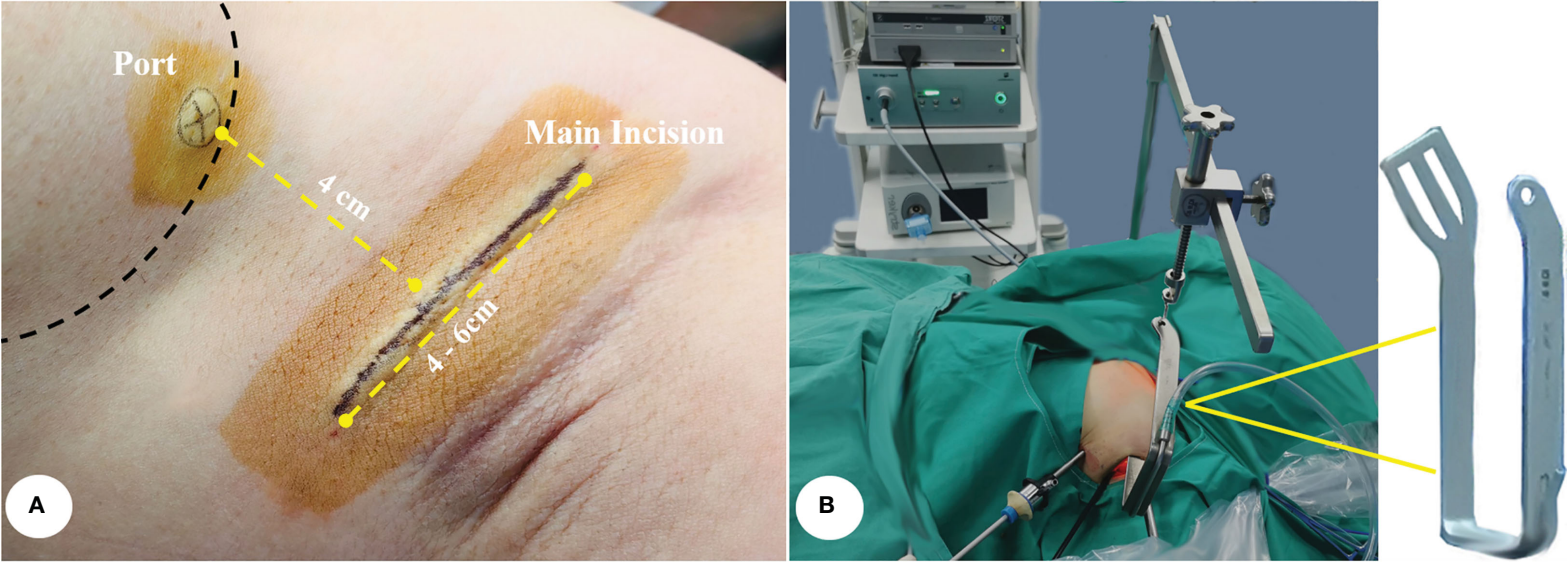
**(A)** The site of main incision and port. **(B)** The retractor that was used to maintain the working space.

The main incision was approximately 4–6 cm along the natural folds in the armpit. Electrocautery was used to incise the subcutaneous fatty layer until the lateral edge of the pectoralis major muscle was exposed and dissected upon the anterior surface of the muscle toward the anterior neck area until the sternocleidomastoid muscle was exposed. A 5 mm port was fixed at the intersection of the lateral margin of the breast and the axillary front line, which was 4 cm away from the middle of the main incision. A right angle retractor was then placed to maintain the working space, and all following procedures were performed with the assistance of an endoscopic camera. The most commonly used endoscopic system was a two-dimensional imaging system equipped with a 30 degree, 10 mm-in-diameter endoscopic lens (**Figure 2**).

#### Key points for the camera holder

Since the majority of TGETs are performed with the assistance of an endoscopic camera, how the camera holder assists during the operation is a critical factor that determines the safety and efficiency of the surgery. Therefore, we summarized three key points for camera holders: one pivot, two positions, and three reference planes.

**Figure 3:**
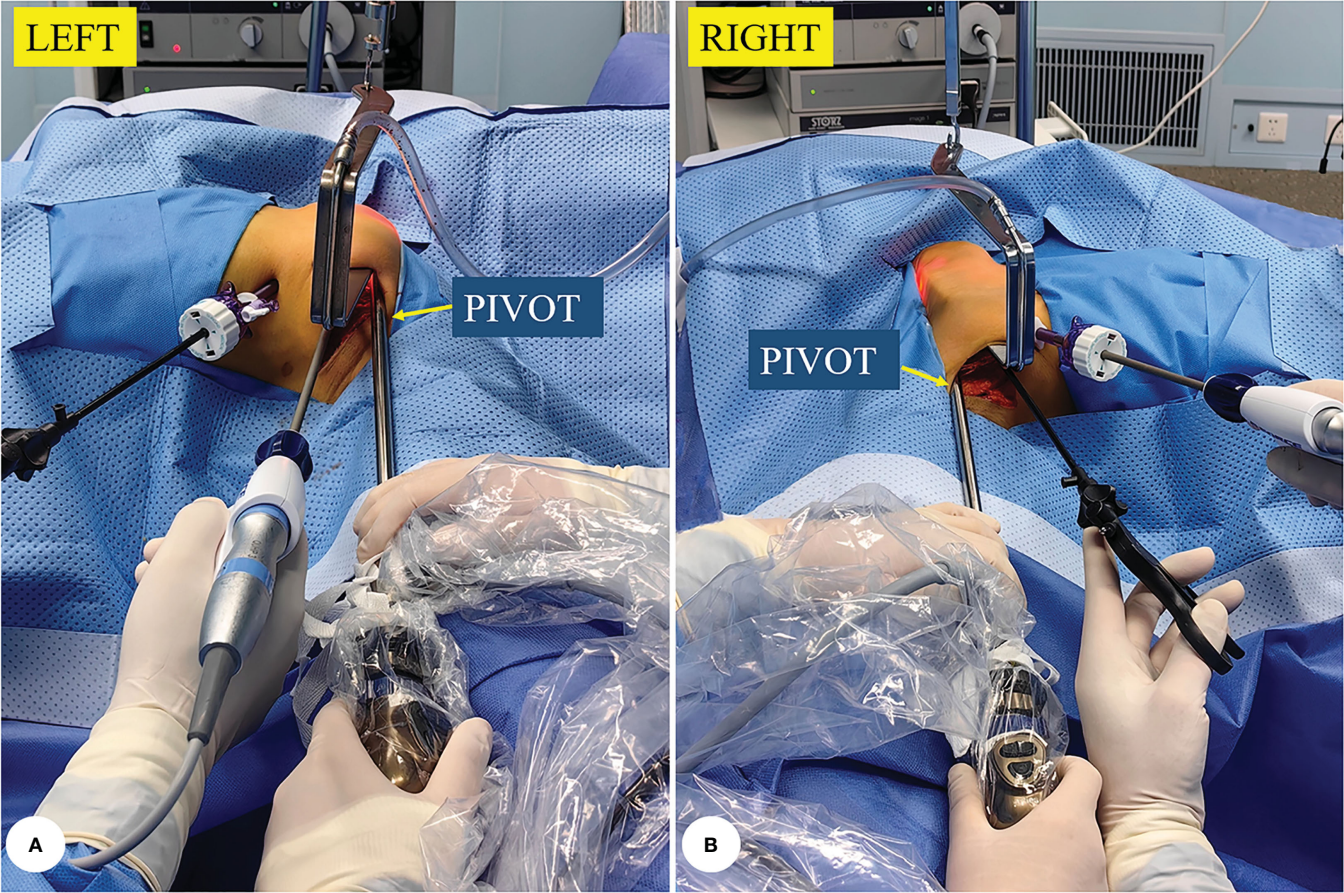
The site of “one pivot” from the left side **(A)** or the right side **(B)**.

“One pivot” means the surface of the pectoralis major muscle. The endoscopic lens should be placed on the surface of the pectoralis major muscle as a pivot during the operation; this not only increases the stability of the camera but also enables the lens and surgical instruments to be located on different planes to avoid interference with each other (**Figure 3**).

**Figure 4:**
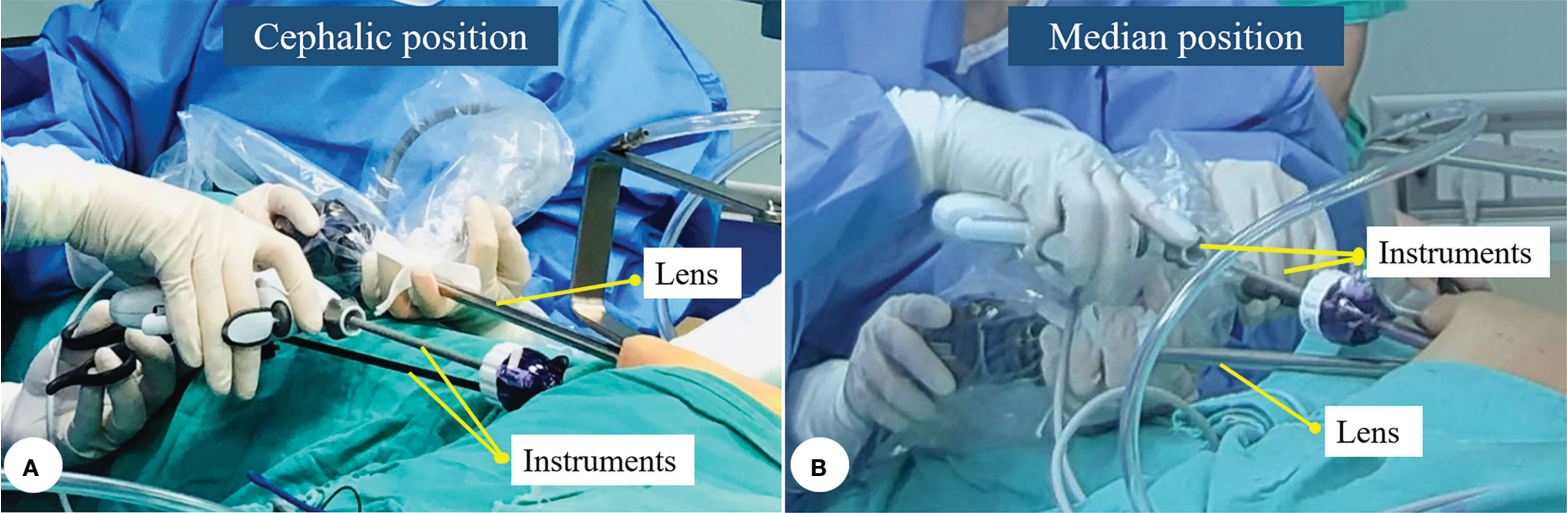
The endoscopic lens was placed in the cephalic **(A)** or median position **(B)**.

“Two positions” refers to placing the endoscopic lens in the cephalic or median position in the working space. One constraint of TGET is the shared working space between the endoscopic lens and surgical instruments. When assisting the surgeon, the camera holder can place the lens in the cephalic or median position according to the habits of each operating group (**Figure 4**).

By placing it in the cephalic position, instrument interference with the lens can be minimized, which provides the best possible viewing experience. However, camera holders need sufficient experience to master this position because it is harder to cover particular areas, such as the superior pole of the thyroid. Alternatively, placing the lens in the median position is much easier to manage. The lens is placed between the surgeon’s two instruments throughout the procedure, which enables it to follow the surgeon’s view and operation simultaneously. However, this view is quite unstable due to interference between the lens and surgical instruments. The following content is based on the cephalic position.

**Figure 5:**
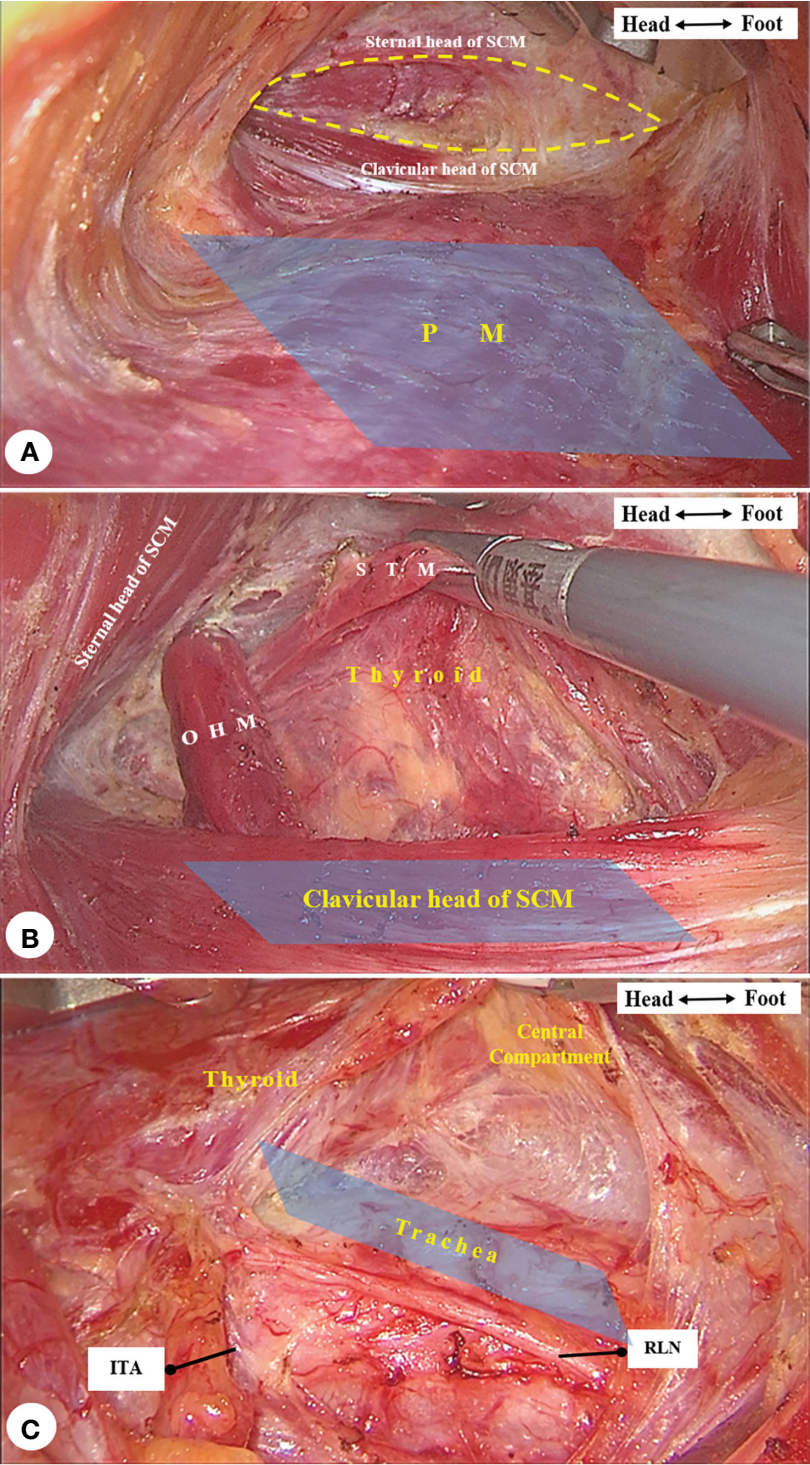
**(A)** The view was parallel to the pectoralis major (PM, marked in blue) while expanding the space (circled in dotted curve) between the two heads of sternocleidomastoid muscle (SCM). **(B)** The view was parallel to the clavicular head of SCM (marked in blue) when releasing the omohyoid muscle (OHM) and exposing the sternothyroid muscle (STM) and thyroid gland. **(C)** The view was parallel to the trachea (marked in blue) with the cephalic side slightly elevated for all following procedures.

“Three reference planes” are planes parallel to the pectoralis major, the clavicular head of the sternocleidomastoid muscle, and the trachea. Since the signs and relationships of anatomic structures look very different in an endoscopic view, we summarized three reference planes following the process of the operation in order to identify certain structures as quickly and accurately and as possible. First, the view should be parallel to the pectoralis major when starting to expand the working space. With the forward depth of space expansion, the view will then be parallel to the clavicular head of the sternocleidomastoid muscle, where exposure of the sternothyroid muscle, thyroid gland, and cervical sheath can be completed. After mobilizing the retractor under the sternothyroid muscle, the whole working space is fully expanded. All the following procedures need delicate operations, and the view should be parallel to the trachea, with the cephalic side slightly elevated approximately 30 degrees. Camera holders should fix the camera at a proper position and adjust the viewing angle of the lens by tilting the light cord connector (**Figure 5**).

#### SGA during thyroidectomy and central neck dissection

The operational scene is posterior to the sternothyroid muscle, anterior to the prevertebral fascia, inferior to the thyroid cartilage, and superior to the margin of the sternum. We separated the operation into five parts and standardized the camera’s view angle for each part.

**Figure 6:**
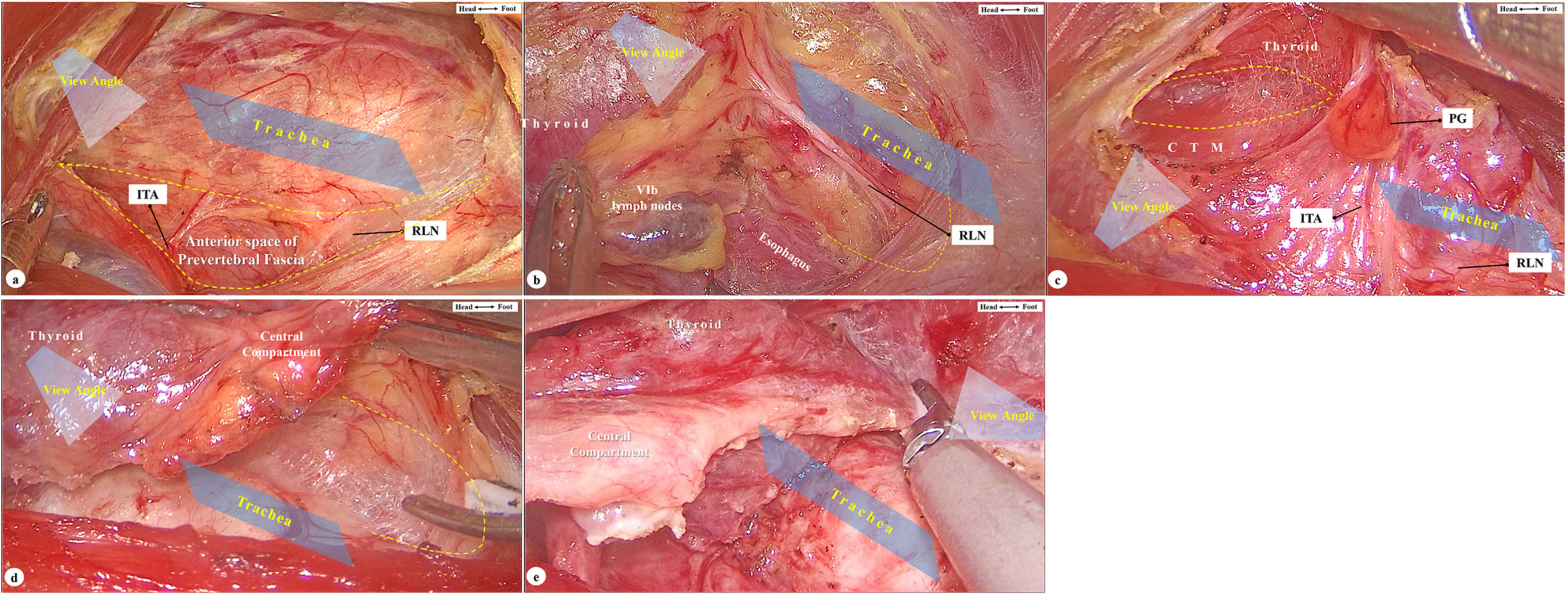
The view angle (marked in light blue), reference plane (marker in dark blue) **(A)**, and anatomic structures. **(A)** Expand the anterior space of the prevertebral fascia (circled in dotted curve) and locate the inferior thyroid artery (ITA) and recurrent laryngeal nerve (RLN). **(B)** Dissect the posterior and inferior (traced with dotted curve) boundary of central compartment. **(C)** Expand the cricothyroid space (circled in dotted curve), dissect the upper pole of the thyroid, and protect the parathyroid gland (PG) in situ. **(D)** Fully dissect the boundary of the thyroid and central compartment (traced with dotted curve). **(E)** Complete thyroidectomy and central neck dissection.

1. Expand the anterior space of the prevertebral fascia.
  View angle: superior and anterior to the surgical instruments, tilting the angles as the surgeon requires.
  Operations: Ligate the middle thyroid vein, locate the inferior thyroid artery at its origin from the internal carotid artery, and separate medially into the anterior space of the prevertebral fascia, which is the posterior boundary of the central neck compartment. Expand the space from the sternal attachment of the sternothyroid muscle to the dorsal side of the upper pole of the thyroid. Present a long-view shot at this part in order to cover the whole operational scene and avoid the contamination caused by activating energy tools (**Figure 6A**).
2. Dissect the posterior boundary of the “mesothroid”.
  Our previous study presented the concept of mesothroid, which is a mesentery-like structure bounded anteriorly by the superficial layer of the deep cervical fascia, posteriorly by the prevertebral fascia, laterally by the carotid arteries, and inferiorly by the innominate artery (6). At this point:
  View angle: inferior and anterior to the surgical instruments, tilting the angles as the surgeon requires.
  Operations: Follow the course of the inferior thyroid artery to the intersection between the artery and the recurrent laryngeal nerve (RLN), coagulating small branches of the vessels nearby. After locating the RLN, carefully dissect along the surface of the RLN until the nerve is fully exposed from where it enters the larynx to the suprasternal fossa. Use intraoperative neuromonitoring, if possible, to avoid injury to the RLN. On the right side, remember to dissect the lymph nodes (VIb) behind the RLN, if necessary. Present a close-up shot when operating along the RLN in order to assist the surgeons’ delicate operations (**Figure 6B**).
3. Dissect the upper pole of the thyroid.
  View angle: superior and posterior to the surgical instruments, tilting the angles as the surgeon requires.
  Operations: Expand the cricothyroid space at the dorsal side of the upper pole of the thyroid, identify and individually ligate the superior thyroid vessels, and avoid injury to the external branch of the superior laryngeal nerve while operating. Identify the superior parathyroid gland in this area and protect the parathyroid gland together with its blood supply as much as possible. Expose and protect the RLN where it reaches the root of the thyroid. The operational space is narrow at this part, camera holders need to present a long-view shot to minimize the influence of energy tools, except a close-up shot would be needed when dealing with the root of the thyroid (**Figure 6C**).
4. Fully dissect the boundary of the thyroid and central compartment.
  View angle: superior and anterior to the surgical instruments, tilting the angles as the surgeon requires.
  Operations: Expand the anterior tracheal space at the dorsal side of the thyroid gland and central neck compartment, superior to the pyramidal lobe and prelaryngeal lymph node and inferior to the suprasternal fossa. Separate the thyroid gland and central lymphatic adipose tissue from the sternothyroid muscle and then sever the inferior boundary of the central neck compartment. Identify and protect the inferior parathyroid gland with its blood supply and preserve the thymus *in situ* to avoid possible injury to the parathyroid gland within. Present a long-view shot with a larger angle since the operation is mainly at the upper half part of the scene (**Figure 6D**).
5. Complete thyroidectomy and central neck dissection.
  View angle: posterior and anterior to the surgical instruments, tilting the angles as the surgeon requires.
  Operations: Harvest the central lymphatic adipose tissue along the medial side of the contralateral sternothyroid muscle, sever the isthmus of the thyroid, and remove the pyramidal lobe and prelaryngeal lymph nodes all together. If the parathyroid glands cannot be preserved in situ, autotransplantation into the pectoralis major muscle should be performed as soon as possible. Present a long-view shot and elevate the cephalic side about 40 degrees to assist the surgeons’ operation on the pyramidal lobe and prelaryngeal lymph nodes (**Figure 6E**).

The TGET procedures were similar to the TGET-SGA procedures; the difference was that the camera holder had no routine guidance during TGET but only followed the instructions as requested by the surgeon.

### Case-control study

We retrospectively reviewed 416 patients who underwent TGET performed by a single surgeon at Nanfang Hospital of Southern Medical University from January 2021 to June 2022. Regarding the application of SGA as a variate, we divided these operations into a TGET group (n = 183) and a TGET-SGA group (n = 233). The demographic characteristics, operative outcomes, and pathological outcomes were analyzed between groups.

### Statistical analysis

Continuous variables were expressed as mean ± standard deviation (SD). Data were analyzed using Student’s t-test and one-way ANOVA. Dichotomous data were compared using chi-squared tests and Fisher’s exact tests. A two-tailed *P*-value < 0.05 was considered statistically significant. The analyses were performed using SPSS version 19.0 software (IBM Corp., Armonk, NY, USA).

## Results

From January 2021 to June 2022, a total of 416 consecutive TGET were performed by a single surgeon at Nanfang Hospital of Southern Medical University. SGA was routinely performed during TGET operations beginning in September 2021. In the current study, 183 patients were enrolled in the TGET group and 233 in the TGET-SGA group.

As listed in **Table 1**, the two groups were similar in terms of age, sex ratio, height, weight, tumor size, Hashimoto’s thyroiditis ratio, and cN1 ratio. The body mass index (BMI) in the TGET-SGA group (22.62 ± 3.48 kg/m^2^) was slightly higher than that in the TGET group (21.81 ± 2.77 kg/m^2^, *P = 0.008*).

**Table 1:** The demographic characteristics of TGET and TGET-SGA group.

Demographic characteristics	TGET group (n=183)	TGET-SGA group (n=233)	P ^a^
Age (Mean ± SD)	35.72 ± 8.72	37.52 ± 9.96	0.050
Gender (male/female, n)	37/146	43/190	0.653
Height (cm)	162.68 ± 7.06	161.67 ± 7.27	0.152
Weight (kg)	57.84 ± 9.20	59.33 ± 11.20	0.139
BMI ^b^	21.81 ± 2.77	22.62 ± 3.48	0.008
Tumor size(cm)	0.79 ± 0.34	0.77 ± 0.28	0.509
Hashimoto’s thyroiditis (n, %)	30, 16.39%	53,22.75%	0.103
cN1 ^c^ (n, %)	42, 23.0%	62, 26.6%	0.392

a P-values <0.05.

b Body Mass Index.

c The preoperative lymph node status was only metastasis to unilateral level VI lymph nodes.

The mean operation time was significantly shorter in the TGET-SGA group (101.82 minutes vs. 114.43 minutes in the TGET group, P < 0.001). The postoperative hospital stay was also shorter in the TGET-SGA group (2.16 days vs. 3.16 days in the TGET group, P < 0.001).

The account of retrieved lymph nodes in the TGET-SGA group was greater than that in the TGET group (6.57 vs. 5.61, P = 0.038). There was similarity between groups in the ratio of patients with metastatic lymph nodes (38.2% in the TGET-SGA group vs. 35.5% in the TGET group) (**Table 2**).

**Table 2:** The operative and pathological outcomes of TGET and TGET-SGA group.

Outcomes	TGET group (n=183)	TGET-SGA group (n=233)	P ^a^
Operation time (mins)	114.43 ± 17.20	101.82 ± 19.39	<0.001
Post-operative Hospital Stay (days)	3.16 ± 0.77	2.16 ± 0.55	<0.001
Retrieved lymph nodes (n)	5.61 ± 4.27	6.57 ± 4.96	0.038
Patients with metastasis lymph nodes (n, %)	65, 35.5%	89,38.2%	0.610

a P-values <0.05.

## Discussion

There are various approaches for performing endoscopic thyroidectomy (3); TGET, a non-insufflation technique, has been widely used in Asia. Many studies have proven its safety and efficacy in low-risk differentiated thyroid cancer (7). A retrospective study including 4129 patients showed that the average operation time was 101.2 ± 38.3 minutes for endoscopic transaxillary gasless thyroidectomy, and the number of harvested lymph nodes was 5.5 ± 4.2 (8). These two indexes were comparable to those found in our study, which proves the stability and reliability of our data. Interestingly, we also discovered that the postoperative hospital stay in TGET-SGA group was also shorter than that in TGET group. It may because of that postoperative hospital stay depends mainly on the nature and amount of fluid drainage after surgery. TGER-SGA group may present a shorter hospital stay due to a less amount of postoperative fluid drainage as a result of less trauma based on a better view during surgery. However, it is still necessary to by verified by a large-scale study.

TGET is a cooperative operation that requires teamwork from at least two people, namely, the surgeon and the camera holder. Timely and accurate camera adjustments can influence the operation time and surgical accuracy. In robot-assisted endoscopic surgery, the surgeon can adjust the camera on his or her own (9). However, it is not a routine solution for all situations, given its high cost. In some other types of surgeries, doctors have tried to invent an endoscopic holding arm to assist in the operation, but it is not applicable to all surgeries (10).

TGET has a view from a side angle, which is one major difference from traditional thyroidectomy. The shared working space of the camera and surgical instruments is another challenge in achieving a successful operation. In order to overcome these difficulties and to train beginners, we came up with this SGA approach. Camera holders should refer to one pivot, two positions, and three planes during the entire TGET operation and follow the view angle suggestions at different parts of the operation mentioned in this article. Based on this retrospective study, we consider it to be an improvement on TGET based on the fact that SGA could shorten the operation time and postoperative hospital stay.

Apart from the potential improvement of SGA on TGET, our research has its own limitations. First of all, the study design of one center and one surgeon may cause the risk of bias on the study results. It was currently difficult for a multi-center study because of the idea of SGA was just raised and applied in our center, with not enough broadly introduction to other centers. In order to minimize the influence of different surgeons on operative results, our study only include data from patients who were treated by one surgeon who was the lead surgeon on TGET in our center. Secondly, the SGA has not been widely used in different centers which makes it not convincible of its improvement in TGET in general. We hope that more information would be gathered from other centers in the future in order to test the effectiveness of SGA in TGET. Finally, SGA was more of an improvement on the subject feeling of surgeons, a questionnaire of surgeons’ experiences during operations would be a better supplement for the following research.

Much of the surgical literature discusses surgical technique improvements or instrument revolutions. There is little focus on the impact of assistants on the success of an operation. In traditional thyroid surgeries, assistants participate in traction, suction, washing, and suturing. Alternatively, in endoscopic thyroid surgeries, assistants participate as observers who hold the camera, which determines what the surgeon sees on the monitor. Training the camera holder to participate more effectively in the operation may improve the process of the operation.

## Data availability statement

The raw data supporting the conclusions of this article will be made available by the authors, without undue reservation.

## Ethics statement

The studies involving human participants were reviewed and approved by the Ethics Committee of Nanfang Hospital of Southern Medical University. The patients/participants provided their written informed consent to participate in this study. Written informed consent was obtained from the individual(s) for the publication of any potentially identifiable images or data included in this article.

## Author contributions

All authors made substantive intellectual contributions to this study to qualify as authors. SL conceived of the design of the study. BS modified the design of the study. SL, BS, SY, and JG performed the study, collected the data, and contributed to the design of the study. ZW and ZZ analyzed the data. BS drafted the manuscript. WC and TL edited the manuscript. All authors read and approved the final manuscript. All authors have agreed to beaccountable for all aspects of the work in ensuring that questions related to the accuracy or integrity of any part of the work are appropriately investigated and resolved. All authors contributed to the article.
